# STAT3 promotes NLRP3 inflammasome activation by mediating NLRP3 mitochondrial translocation

**DOI:** 10.1038/s12276-024-01298-9

**Published:** 2024-09-02

**Authors:** Ling Luo, Fupeng Wang, Xueming Xu, Mingliang Ma, Guangyan Kuang, Yening Zhang, Dan Wang, Wei Li, Ningjie Zhang, Kai Zhao

**Affiliations:** 1 Department of Hematology and Critical Care Medicine, Third Xiangya Hospital, Central South University, Changsha, Hunan Province 410000 P PR China; 2 Department of Dermatology, Third Xiangya Hospital, Central South University, Changsha, Hunan Province 410000 P PR China; 3 Department of Rheumatology, First Affiliated Hospital of Zhengzhou University, Zhengzhou, Henan Province 450000 P PR China; 4 Department of Blood Transfusion, Second Xiangya Hospital, Central South University, Changsha, Hunan Province 410000 P PR China; 5 Key Laboratory of Sepsis Translational Medicine of Hunan, Central South University, Changsha, Hunan Province 410000 P PR China

**Keywords:** Cell death and immune response, Cell signalling

## Abstract

Recognition of the translocation of NLRP3 to various organelles has provided new insights for understanding how the NLRP3 inflammasome is activated by different stimuli. Mitochondria have already been demonstrated to be the site of NLRP3 inflammasome activation, and the latest research suggests that NLRP3 is first recruited to mitochondria, then disassociated, and subsequently recruited to the Golgi network. Although some mitochondrial factors have been found to contribute to the recruitment of NLRP3 to mitochondria, the detailed process of NLRP3 mitochondrial translocation remains unclear. Here, we identify a previously unknown role for Signal transducer and activator of transcription-3 (STAT3) in facilitating the translocation of NLRP3 to mitochondria. STAT3 interacts with NLRP3 and undergoes phosphorylation at Ser727 in response to several NLRP3 agonists, enabling the translocation of STAT3 and thus the bound NLRP3 to mitochondria. Disruption of the interaction between STAT3 and NLRP3 impairs the mitochondrial localization of NLRP3, specifically suppressing NLRP3 inflammasome activation both in vitro and in vivo. In summary, we demonstrate that STAT3 acts as a transporter for mitochondrial translocation of NLRP3 and provide new insight into the spatial regulation of NLRP3.

## STAT3 promotes NLRP3 inflammasome activation by mediating NLRP3 mitochondrial translocation

Our bodies have an innate immune system, which is the first line of defense against infections. It uses specific receptors to detect harmful pathogens. This research investigates how a protein, STAT3, affects the activation of a complex, the NLRP3 inflammasome, crucial in our immune response. The study involved experiments with cell cultures and mice, identifying how STAT3 helps move NLRP3 within cells for activation. This was seen in an experimental study. The results show that targeting STAT3 can reduce inflammation caused by the NLRP3 inflammasome. This suggests a new treatment strategy for diseases caused by too much inflammation, like gout, diabetes, and Alzheimer’s disease. The study shows the potential of drugs that stop STAT3 to manage or treat inflammatory conditions, paving the way for future research on such treatments.

This summary was initially drafted using artificial intelligence, then revised and fact-checked by the author.

## Introduction

As the first line of host defense, the innate immune system uses pattern recognition receptors (PRRs) to detect invading pathogens and endogenous cellular damage to activate signaling pathways and maintain homeostasis. Numerous PRRs, including Toll-like receptors (TLRs), RIG‑I‑like receptors (RLRs) and NOD-like receptors (NLRs)^1–3^, have been identified. NLRs are cytosolic PRRs that induce immune responses by forming inflammasomes. Inflammasomes are supramolecular complexes consisting of sensors (NLRs), an adaptor (apoptosis-associated speck-like protein containing a CARD (ASC)) and an executor (caspase-1). Upon activation, the sensors oligomerize and then recruit ASC, which in turn recruits caspase-1 to enable its maturation. Mature caspase-1 is able to convert IL-1 family proteins into their activated forms and cleave gasdermin D (GSDMD) to trigger pyroptosis, which is responsible for inflammatory responses^4–7^.

The most well-studied inflammasome is the NLRP3 inflammasome. The NLRP3 inflammasome can be activated by diverse stimuli, including components of pathogens, environmental particles, and endogenous damage signals. The NLRP3 inflammasome promotes host defense against infections; however, its aberrant activation leads to several metabolic- and aging-associated inflammatory disorders, such as atherosclerosis, gout, diabetes, and Alzheimer’s disease. Notably, NLRP3 gain-of-function mutations cause autoinflammatory cryopyrin-associated periodic syndrome (CAPS). Thus, the NLRP3 inflammasome has attracted much attention because it is a highly relevant target for therapeutic intervention^8–10^.

NLRP3 inflammasome activation is accomplished through a two-step process comprising a priming step and an activation (or assembly) step. The priming signal is provided by pathogen ligands, such as Lipopolysaccharides (LPS), and inflammatory cytokines, such as TNF-α, which can greatly upregulate NLRP3 and IL-1β expression. Priming also mediates posttranslational modifications (PTMs) on NLRP3, which positively or negatively regulate the inflammasome. The activation signal is provided by different stimuli, such as ATP, nigericin, or monosodium urate (MSU), triggering the formation and full activation of the NLRP3 inflammasome^11,12^. Despite several proposed explanations, including potassium efflux, lysosomal disruption, ROS production, metabolic changes and trans-Golgi disassembly, the mechanism through which NLRP3 senses numerous stimuli remains unclear. Recently, the observation of the recruitment of NLRP3 to the Golgi and endosomal network, as well as its previously described recruitment to mitochondria and the endoplasmic reticulum (ER), have provided deep insight into how NLRP3 is activated; that is, NLRP3 needs to be in the “right place” for activation^12^. Via experiments with a live-cell multispectral time-lapse tracking system, a recent study further demonstrated that NLRP3 is translocated to mitochondria at approximately 10–15 min post stimulation^13^ and is then disassociated from mitochondria and subsequently recruited to the Golgi network. This work, in addition to previous studies, have demonstrated that mitochondrial localization is required for NLRP3 activation. Although three factors (cardiolipin, mitochondrial antiviral signaling protein (MAVS), and mitofusin-2)^14–16^ on the surface of mitochondria have been suggested to bind NLRP3 and recruit it to mitochondria, the process by which NLRP3 is transported to mitochondria is not clear.

To clarify the underlying mechanism, we investigated a series of endogenous cellular signals by screening small compounds. Surprisingly, we observed that treatment with an inhibitor of STAT3 decreased the mitochondrial localization of NLRP3 and suppressed NLRP3 inflammasome activation both in vitro and in vivo. STAT3 is a transcription factor that mediates numerous acute and chronic inflammatory processes and has a noncanonical role in regulating the function of the mitochondrial electron transport chain (ETC)^17–19^. Here, we demonstrated that STAT3 promotes the translocation of NLRP3 to mitochondria and its subsequent activation, revealing a new role for STAT3 in the spatial regulation of NLRP3.

## Materials and Methods

### Mice

Wild-type C57BL/6 mice (6–8 weeks old) were purchased from Hunan SJA Laboratory Animal Co., Ltd. (Changsha, China). All the animals were housed under specific pathogen-free (SPF) conditions in the Central South University Animal Facility. The animal experiments were conducted in accordance with the Institutional Animal Care and Use Committee of Central South University (CSU-2022-0657).

### Regents

Small molecule compounds were purchased from Selleck Co. (Cherry Pick Library 96-well-L2000-Z451886-30 uL). The anti-caspase-1 antibody (Abcam, ab179515), anti-IL-1β antibody (RD Systems, AF-401-NA), anti-NLRP3 antibody (AdipoGen, AG-20B-0014-C100), anti-ASC antibody (AdipoGen, AG-25B-0006-C100), anti-β-actin antibody (Cell Signaling Technology, #3700), anti-STAT3 antibody (Cell Signaling Technology, #4904), anti-pY705-STAT3 antibody (Cell Signaling Technology, #9145), anti-pS727-STAT3 antibody (Cell Signaling Technology, #9134), anti-GAPDH antibody (Cell Signaling Technology, #2118), anti-VDAC antibody (Cell Signaling Technology, #4866), anti-LaminA/C antibody (Cell Signaling Technology, #4777), anti-DYKDDDDK tag antibody (Cell Signaling Technology, #2368), and anti-Myc-Tag Antibody (Cell Signaling Technology, #2272) were obtained from the indicated suppliers. Sheep anti-rabbit IgG-h + l DyLight 488 conjugate (BETHY, A120-100D2), Alexa Fluor 594 goat anti-mouse IgG (Biolegend, 405326), and protein A/G-agarose beads (Santa Cruz, sc-2003) were obtained from the indicated suppliers. Anti-Flag affinity gel (Sigma, A2220) and Pierce Anti-c-Myc agarose (Thermo Fisher Scientific, 20168) were obtained from the indicated suppliers. MitoTracker Red CMXRos (Invitrogen, M7512), MitoTracker Deep Red FM (Invitrogen, M22426), a Duolink In Situ Detection Reagents Red Kit (Sigma, DUO92008), Duolink In Situ PLA Probe Anti-Mouse MINUS (Sigma, DUO82004), and Duolink In Situ PLA Probe Anti-Rabbit PLUS (Sigma, DUO82002) were obtained from the indicated suppliers. Cell lysis buffer (Cell Signaling Technology, 9803 S), BCA Protein Assay Kits (Thermo Scientific, 23225), and an LDH Cytotoxicity Assay Kit (Beyotime, C0017) were obtained from the indicated suppliers.

### Cell culture and stimulation of macrophages

Wild-type C57BL/6 mice were injected intraperitoneally with 3% thioglycollate, and three days later, primary macrophages were harvested in RPMI 1640 medium by peritoneal lavage; the purity of the isolated primary macrophages was as high as 95%, as determined by flow cytometry (Supplementary Fig. 1). Primary peritoneal macrophages were seeded into 6-well plates, 24-well plates, or 48-well plates depending on the experiment.

Different agonists and stimuli were used in this study as previously described in ref. ^20^. For NLRP3 inflammasome activation, peritoneal macrophages were primed with LPS (100 ng/mL) for 3 h and then stimulated with ATP (5 mM, 1 h), nigericin (10 μM, 1 h) or MSU (200 μg/mL, 6 h). For AIM2 inflammasome activation, after priming, cells were transfected with poly(dA:dT) (1 μg/mL) using Lipofectamine 3000. For NLRC4 inflammasome activation, primed macrophages were transfected with Flagellin (2 μg/mL) with Lipofectamine 3000 for 1 h.

### Cell lines

HEK293T cells were obtained from the American Type Culture Collection (Manassas, VA) and cultured in DMEM supplemented with 10% fetal bovine serum, 100 U/mL penicillin and 100 μg/mL streptomycin.

### Plasmids and transfection

The NLRP3, caspase-1, pro-IL-1β, ASC and NEK7 plasmids were constructed as previously described in ref. ^20^. The full-length sequence of STAT3 was amplified from iBMDM cDNA, and the sequences of the primers used were as follows: 5′-AACGGGCCCTCTAGACTCGAG ATGGCTCAGTGGAACCAGCTGCAGCAGCTGGA-3′ and 3′-TAGTCCAGTGTGGTGGAATTC CATGGGGGAGGTAGCACACTCCGAGGTCAGAT-5′. Then, the sequence was cloned and inserted into pcDNA3.1 vectors that contained different tags. Then, the plasmids were transiently transfected into HEK293T cells with linear polyethylenimine at a 1:3 mass:volume ratio. The cell culture medium was changed 6–8 h after transfection, and culture was continued. Then, the cells were collected 18–24 h later for Western blot analysis.

### Immunoprecipitation and western blot analysis

Peritoneal macrophages stimulated as indicated or transfected HEK293T cells were lysed in cold IP buffer containing 50 mM Tris HCl (pH 7.4), 50 mM EDTA, 200 mM NaCl, 1% NP-40 and a protease inhibitor cocktail (Roche, 11873580001). The macrophage lysate was incubated with a primary antibody at 4 °C overnight to allow the formation of antigen-antibody complexes. The formed antigen-antibody complexes were coincubated with protein A/G-agarose beads at 4 °C for 2 h, washed three times with IP buffer, and then eluted in loading buffer. The HEK293T cell lysates were directly incubated with anti-Flag affinity gel or anti-c-Myc agarose at 4 °C for 2 h, after which the gel/agarose was washed 5 times with IP buffer and the complexes were eluted in loading buffer. The eluted samples were subjected to immunoblot analysis.

For immunoblot analysis, stimulated macrophages were lysed in cell lysis buffer supplemented with protease inhibitor cocktail and the phosphatase inhibitor PMSF. The protein concentration after lysis was determined by a BCA protein assay kit. The quantified proteins were separated by sodium dodecyl sulfate‒polyacrylamide gel electrophoresis and then transferred onto a 0.2 µM nitrocellulose membrane for immunoblot analysis.

### Small interfering RNA transfection

For siRNA-mediated silencing of STAT3, cells were cultured in 24-well plates (2 × 10^5^ cells per well) or 6-well plates (9 × 10^5^ cells per well), and the siRNAs were then transfected with RNAiMAX Transfection Reagent (Invitrogen, 13778) following the manufacturer’s instructions. Seventy-two hours after transfection, the cells were stimulated with different inflammasome agonists. The siRNA target sequence was 5′- GCUGAAAUCAUCAUGGGCUAUTT -3′, and the scrambled negative control siRNA sequence was 5′- UUCUCCGAACGUGUCACGU-3′. The silencing efficiency was examined by western blotting using the corresponding antibodies. The indicated scrambled siRNAs were chemically synthesized by Sangon Biotech (Shanghai) Co., Ltd.

### Quantitative PCR

RNA was extracted using an RNA Fast 200 Kit (Fastagen, 22001). Complementary DNA was synthesized by using TransScript All-in-One First-Strand cDNA Synthesis SuperMix for qPCR (TransGen Biotech) according to the manufacturer’s protocols. Quantitative PCR was performed using SYBR Green (Vazyme Biotech) on a LightCycler 480 instrument (Roche Diagnostics), and the expression level of each target mRNA was individually normalized to that of β-actin. The sequences of the q-PCR primers used in this study are listed in the table below:

**Table:** 

Gene Name	Primer Direction	Sequence
NLRP3	Forward	TGGATGGGTTTGCTGGGAT
	Reverse	CTGCGTGTAGCGACTGTTGAG
STAT3	Forward	CAATACCATTGACCTGCCGAT
	Reverse	GAGCGACTCAAACTGCCCT
β-actin	Forward	AGTGTGACGTTGACATCCGT
	Reverse	GCAGCTCAGTAACAGTCCGC
pro-IL-1β	Forward	GCAACTGTTCCTGAACTCAACT
	Reverse	ATCTTTTGGGGTCCGTCAACT

### ASC speck formation assay

To evaluate ASC speck formation, peritoneal macrophages were seeded on chamber slides and allowed to attach overnight. The following day, the cells were primed with LPS and treated with nigericin in the presence or absence of the indicated inhibitors. Then, the cells were fixed with 4% paraformaldehyde in PBS for 15 min and washed three times in PBS with Tween 20 (PBST) prior to permeabilization with 0.1% Triton X-100 for 10 min. After blocking with 3% bovine serum albumin in PBS for 1 h, the cells were incubated with primary antibodies overnight at 4 °C. After washing with PBST, the cells were incubated with secondary antibodies in 3% bovine serum albumin for 30 min, and nuclei were then stained with DAPI (Beyotime, P0131). The cells were visualized by fluorescence microscopy (Nikon Ti2-U).

### Proximity ligation assay

Proximity ligation assays were performed using Duolink reagents (Sigma) according to the manufacturer’s instructions to visualize the interaction between the STAT3 and NLRP3 proteins and their localization in mitochondria-resident mouse peritoneal macrophages. To study the interaction between STAT3 and NLRP3, cells were grown on PTFE printed microscope slides (Electron Microscopy Science, 63423-08) and subjected to canonical stimulation of the NLRP3 pathway in the presence or absence of the mitochondrial dye MitoTracker Deep Red FM. The cells were fixed, permeabilized, blocked, and then incubated with primary antibodies overnight at 4 °C. After incubation with the primary antibodies, the cells were incubated with a combination of the corresponding PLA probes and secondary antibodies conjugated to oligonucleotides for 1 h at 37 °C. After washing with buffer A [0.01 M Tris-HCl (pH 7.4), 0.15 M NaCl, 0.05% Tween 20], the cells were incubated with ligation mix (Sigma, DUO92008) for 30 min at 37 °C to allow the formation of a closed circle DNA template when the PLA probes were bound in close proximity. After washing with buffer A, the cells were incubated with polymerase mix (Sigma, DUO92008) for 100 min at 37 °C to allow rolling circle amplification. After sequential washes with buffer B [0.2 M Tris-HCl (pH 7.4), 0.1 M NaCl] and buffer C (10-fold dilution of buffer B with water), cover slips (Citotest, 10212450 C) were mounted onto the microscopy slides. The cells were imaged using a 63×/1.4 oil immersion objective on a Leica STELLARIS 5 confocal microscope (Leica).

### Immunofluorescence staining and confocal microscopy

Primed cells were treated with DMSO or with napabucasin followed by nigericin for 30 min. At the same time, the cells were stained for 30 min at 37 °C with 100 nM MitoTracker Red CMXRos (Invitrogen, M7512) in the dark. After washing three times with warm PBS, the cells were fixed with 4% paraformaldehyde in PBS for 15 min and permeabilized with 0.1% Triton X-100 for 10 min. After blocking with 3% bovine serum albumin in PBS for 1 h, the cells were incubated overnight at 4 °C with primary antibodies (anti-NLRP3 and anti-STAT3 antibodies). After washing with PBST, the cells were incubated with Sheep anti-rabbit IgG-h + l DyLight 488 conjugate and Alexa Fluor 594-conjugated goat anti-mouse IgG in PBS containing 3% BSA for 30 min and rinsed in PBST. Nuclei were stained with DAPI (Beyotime, P0131). Images were analyzed by using a 63×/1.4 oil immersion objective on a Leica STELLARIS 5 confocal microscope (Leica).

### Cellular thermal shift assay (CETSA)

Peritoneal macrophages were collected and resuspended at a density of 4 × 10^5^ cells/mL in a 1.5 mL Eppendorf tube and were then washed with ice-cold PBS. The macrophages were resuspended in RIPA lysis buffer containing the protease inhibitor cocktail. The cell lysates were centrifuged at 12,000 × g for 15 min at 4 °C, after which the supernatants were collected. The lysate supernatants were incubated with napabucasin (20 μM) or the control (DMSO) for 1 h at room temperature. Then, each drug-treated cell lysates was aliquoted into PCR tubes (60 μL each), and the tubes were heated at different temperatures (37, 47, 57, 67, 77, and 87 °C) for 5 min in the PCR apparatus and then cooled at room temperature for 3 min. The soluble fractions were separated after centrifugation of the lysates at 20,000 × g for 20 min at 4 °C, transferred to new microcentrifuge tubes, and analyzed by SDS‒PAGE followed by western blotting with anti-NLRP3 and anti-STAT3 antibodies.

### Separation of the cytoplasmic and nuclear fractions

The cytoplasmic and nuclear fractions were separated from lysates of peritoneal macrophages using a cytoplasmic and nuclear fractionation kit (Beyotime, P0028) according to the manufacturer’s guidelines. Then, the isolated protein components were quantified by a BCA assay and analyzed by immunoblotting.

### ELISA and LDH assay

Supernatants from cell cultures and sera were analyzed using IL-1β and TNF-a ELISA kits (Invitrogen, 88-5019-88, 88-7324-88) according to the manufacturer’s instructions. Cell death was assessed with an LDH Cytotoxicity Assay Kit (Beyotime, C0017) according to the manufacturer’s instructions.

### In vivo LPS challenge

Wild-type C57BL/6 male mice were injected with saline or napabucasin (5 mg/kg) half an hour before injection with LPS (20 mg/kg body weight) and were then monitored for 8 h. The mice were deeply anesthetized with sodium pentobarbital (70 mg/kg, i.p.), and the cardiac blood and left lung were collected from each mouse. Then, the serum concentrations of IL-1β and TNF-α were measured by ELISA, and lung damage was evaluated by HE staining.

### MSU-induced peritonitis modeling in vivo

Wild-type C57BL/6 male mice were intraperitoneally injected with saline or napabucasin (5 mg/kg), half an hour before injection of 2 mg of MSU (dissolved in 300 μL of saline) and were then monitored for 6 h. After the mice were killed, the peritoneal lavage fluid was collected by lavage of the peritoneal cavity with ice-cold PBS; the lavage fluid was concentrated for ELISA, and neutrophils were counted.

### Statistical analysis

The data were analyzed using GraphPad Prism software (version 9.5.0). The data are presented as the standard deviation of the mean (SD) or standard error of the mean (SEM), depending on the experiment. Independent sample t-tests and two-way ANOVA followed by the Bonferroni correction were performed in this study. Differences with a *p*-value < 0.05 were considered to be statistically significant, and the details of the statistical analyses can be found in the figure legends. In the animal studies, the mice were randomly divided into different groups, and all the samples were processed in a blinded manner. For western blotting, qPCR and other quantitative methods, experiments were performed at least three times.

## Results

### Identification of STAT3 inhibitors that regulate the NLRP3 inflammasome

**Fig. 1:**
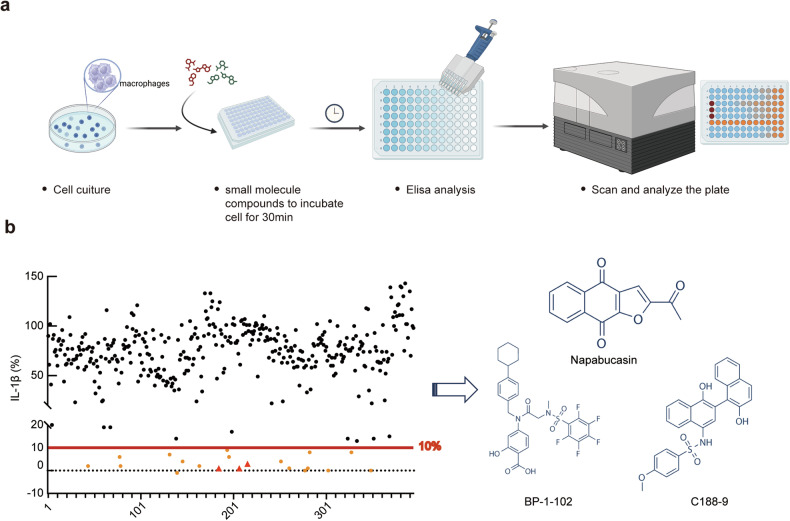
Identification of STAT3 inhibitors that regulate the NLRP3 inflammasome. **a** Screening for small molecule compounds obtained from Selleck Co. This figure template, “Screening flow chart,” was assembled using dynamic BioRender assets (icons, lines, shapes and/or text) and is fully editable. **b** Percentage inhibition of IL-1β secretion by each compound. The IL-1β secretion level relative to 10% of that in the control group was used as a cutoff (left), and the chemical structures of the compounds napabucasin, BP-1-102 and C188-9 used to inhibit STAT3 are shown (right).

To elucidate whether endogenous cellular signals are involved in NLRP3 translocation, we detected IL-1β secreted in response to treatment with NLRP3 agonists (LPS+nigericin) and screened 395 small compounds obtained from Selleck Co. (Supplementary Table 1) for their ability to inhibit IL-1β secretion (Fig. 1a). These compounds were inducers or inhibitors of numerous intrinsic signaling pathways, including the MAPK, PI3K/AKT/mTOR, JAK-STAT, TGF-β-Smad, and integrin signaling pathways. We treated macrophages with the compounds after treatment with LPS to focus on regulation at the NLRP3 inflammasome activation stage rather than the priming stage (Fig. 1a). Intriguingly, 20 compounds inhibited IL-1β secretion by at least 90%, and three of them, namely, napabucasin, BP-1-102 and C188-9, attracted our attention (Fig. 1b) since they are inhibitors of STAT3.

### STAT3 promotes NLRP3 inflammasome activation in both transcription-dependent and transcription-independent manners

**Fig. 2:**
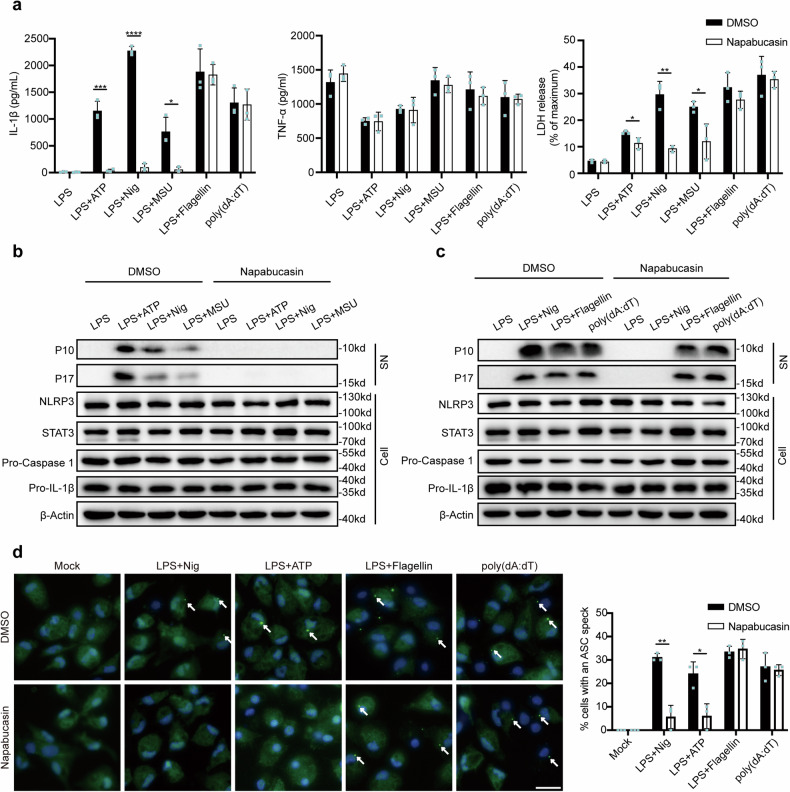
Napabucasin specifically suppresses NLRP3 inflammasome activation. **a** ELISA of IL-1β and TNF-α secretion and assay of LDH release in supernatants from LPS-primed mouse peritoneal macrophages treated with 10 μM napabucasin for 30 min followed by the indicated stimulators of different inflammasomes. The experiments were repeated at least three times, and representative data are shown. **b**, **c** Immunoblot analysis of supernatants (SN) or cell lysates (Cell) from mouse peritoneal macrophages treated with the indicated stimulators of the NLRP3 (**b**), NLRC4 or AIM2 inflammasome with or without napabucasin (**c**). **d** Representative images of ASC specks in peritoneal macrophages treated with the indicated stimuli; ASC, green; nuclei, blue. The white arrows indicate ASC specks. Scale bar: 20 μm. The percentage of cells containing an ASC speck was quantified (right). At least 100 peritoneal macrophages from each genotype were analyzed. The results are presented as the means ± SDs, and representative photographs from three biologically independent experiments with similar results are shown. Statistical analyses were carried out via two-way ANOVA with the Bonferroni correction in (**a**, **d**). *P < 0.05, **P < 0.01 and ***P < 0.001.

To further confirm the function of STAT3 inhibitors in inflammasome activation, we treated macrophages with nigericin, ATP and MSU, which activate the NLRP3 inflammasome via different mechanisms, as well as with flagellin and poly(dA:dT), which activate the NLRC4 and AIM2 inflammasomes, respectively. Given that napabucasin has been applied in clinical trials, we chose to use napabucasin for follow-up experiments^21,22^. We observed that napabucasin greatly inhibited IL-1β secretion but not TNF-α secretion in response to stimulation of the NLRP3 inflammasome, although it had no effect on the NLRC4 or AIM2 inflammasome. Accordingly, lactic acid dehydrogenase (LDH) release triggered by NLRP3 stimulation was also impaired by napabucasin treatment (Fig. 2a). This observation was also verified by the detection of cleaved caspase-1 (p10) and pro-IL-1β (p17) in the supernatants of macrophages treated with these agonists (Fig. 2b, c). We also noted that napabucasin barely affected the protein levels of NLRP3 and pro-IL-1β after LPS priming (Fig. 2b, c), excluding its role in the regulation of the NF-κB pathway. ASC speck formation is a hallmark of NLRP3 inflammasome assembly and can be monitored by immunofluorescence staining. We further observed that napabucasin inhibited ASC speck formation in ATP- and nigericin-treated macrophages but not in flagellin- or poly(dA:dT)-treated macrophages (Fig. 2d). Thus, these results demonstrate that STAT3 inhibitors specifically suppress the activation of the NLRP3 inflammasome, suggesting that STAT3 may be involved in the regulation of NLRP3 inflammasome assembly via an unknown mechanism.

**Fig. 3:**
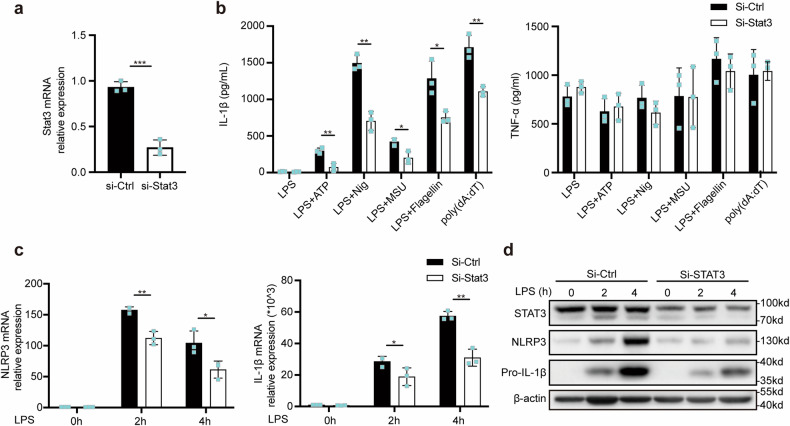
STAT3 promotes NLRP3 inflammasome activation at the transcriptional level. **a** Quantitative PCR analysis of STAT3 mRNA expression in mouse peritoneal macrophages after transfection with NC-siRNA or STAT3-siRNA for 48 h. **b** IL-1β and TNF-α secretion in supernatants from mouse peritoneal macrophages transfected as described in (**a**) and then treated with the indicated stimuli. **c**, **d** Relative NLRP3 and IL-1β mRNA expression (**c**) in peritoneal macrophages treated with LPS for 2 h and 4 h or not treated after transfection as described in (**a**). Target mRNA expression was normalized to the expression of β-actin as the reference gene. Immunoblot analysis of NLRP3 and Pro-IL-1β expression in mouse peritoneal macrophages transfected as described; the photographs are representative of three biologically independent experiments with similar results (**d**). The results are presented as the means ± SDs; *n* = 3 biologically independent experiments (**a**–**c**). Statistical analyses were carried out via independent sample t-test in (**a**) or two-way ANOVA with the Bonferroni correction in (**b**, **c**). *P < 0.05, **P < 0.01 and ***P < 0.001.

Next, we examined the role of STAT3 in the regulation of the NLRP3 inflammasome. STAT3 was knocked down in mouse primary macrophages by siRNA transfection (Fig. 3a), and silencing STAT3 indeed suppressed IL-1β secretion in macrophages treated with NLRP3, NLRC4, or AIM2 agonists (Fig. 3b). We found that silencing STAT3 also decreased NLRP3 and pro-IL-1β expression at the translational and transcriptional levels^23–25^ (Fig. 3c, d), in contrast to the results obtained with the STAT3 inhibitor. These effects of STAT3 silencing were consistent with those observed in previous studies indicating that STAT3 could promote NLRP3 and IL-1β expression at the transcriptional level. Taken together, these findings indicate that either knockdown of STAT3 or blockade of STAT3 by treatment with inhibitors can suppress NLRP3 inflammasome activation via different mechanisms.

### Cytoplasmic STAT3 interacts with NLRP3

**Fig. 4:**
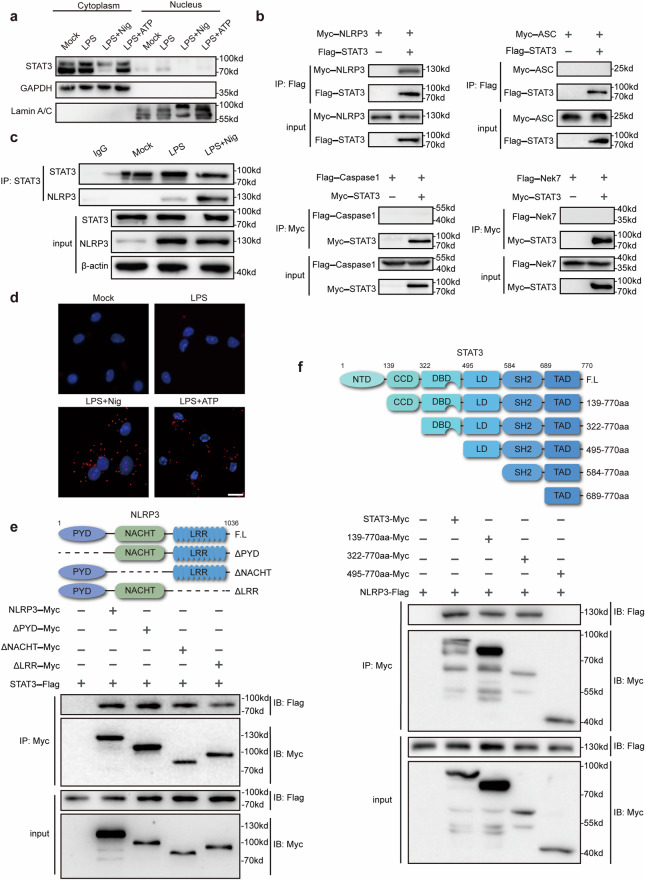
Cytoplasmic STAT3 interacts with NLRP3. **a** Immunoblot analysis of cytoplasmic and nuclear components after treatment with the corresponding stimuli described above. **b** Immunoprecipitation and immunoblot analysis of lysates from HEK293T cells transfected with Flag-STAT3, Myc-NLRP3, Myc-ASC, Myc-STAT3, Flag-caspase-1, or Flag-Nek7. Immunoprecipitation was performed with anti-Flag (up) and anti-Myc (down) antibodies, and immunoblotting was performed with anti-Myc and anti-Flag antibodies, respectively. **c** Immunoblot analysis of lysates from mouse peritoneal macrophages treated with the indicated stimuli. IP was performed with an anti-STAT3 antibody, and immunoblotting was then performed with an anti-NLRP3 antibody. **d** The physical interaction between NLRP3 and STAT3 was visualized as red puncta by a PLA in mouse peritoneal macrophages primed with 100 ng/mL LPS for 3 h and then stimulated with nigericin (10 μM) or ATP (5 mM) for 1 h. Scale bar: 20 μm. **e** Myc-tagged NLRP3 or its mutants and Flag-STAT3 were cotransfected into HEK293T cells for 24 h prior to immunoprecipitation with anti-Myc beads and western blotting (bottom). Schematic diagram of NLRP3 and its truncation mutants (top). **f** Myc-STAT3 or its mutants and Flag-NLRP3 were cotransfected into HEK293T cells for 24 h prior to immunoprecipitation with anti-Myc beads and western blotting (bottom). Schematic diagram of STAT3 and its truncation mutants (top). The results are presented as the means ± SDs, and representative photographs of three biologically independent experiments with similar results are shown.

To further study the nontranscriptional function of STAT3 in NLRP3 inflammasome activation, we examined the distribution of STAT3 during the process of NLRP3 inflammasome activation. STAT3 translocated to the nucleus upon LPS treatment (Fig. 4a), as suggested by a previous study. Intriguingly, upon the addition of nigericin or ATP, STAT3 was located primarily in the cytoplasm (Fig. 4a), suggesting that cytoplasmic STAT3 may play a role in the assembly of the NLRP3 inflammasome. Therefore, we investigated whether cytoplasmic STAT3 can interact with NLRP3 inflammasome components. By overexpressing STAT3 with NLRP3, ASC, NEK7 or caspase-1 in HEK293T cells, we found that STAT3 could interact with NLRP3 but not with ASC, NEK7 or caspase-1 (Fig. 4b). This interaction was verified by immunoprecipitation in primary macrophages, and nigericin treatment dramatically promoted the association between STAT3 and NLRP3 (Fig. 4c). A PLA (proximity ligation assay) assay was further used to demonstrate this interaction in primary macrophages (Fig. 4d). Moreover, to determine which domains of STAT3 and NLRP3 are required for this interaction, we constructed plasmids expressing different truncation mutants of STAT3 and NLRP3. We observed that the DBD of STAT3 and at least two domains, the PYD and LRR domains, of NLRP3 mediate the interaction between STAT3 and NLRP3 (Fig. 4e, f). Thus, cytoplasmic STAT3 interacts with NLRP3 at the NLRP3 inflammasome activation stage.

### STAT3 transports NLRP3 to mitochondria

**Fig. 5:**
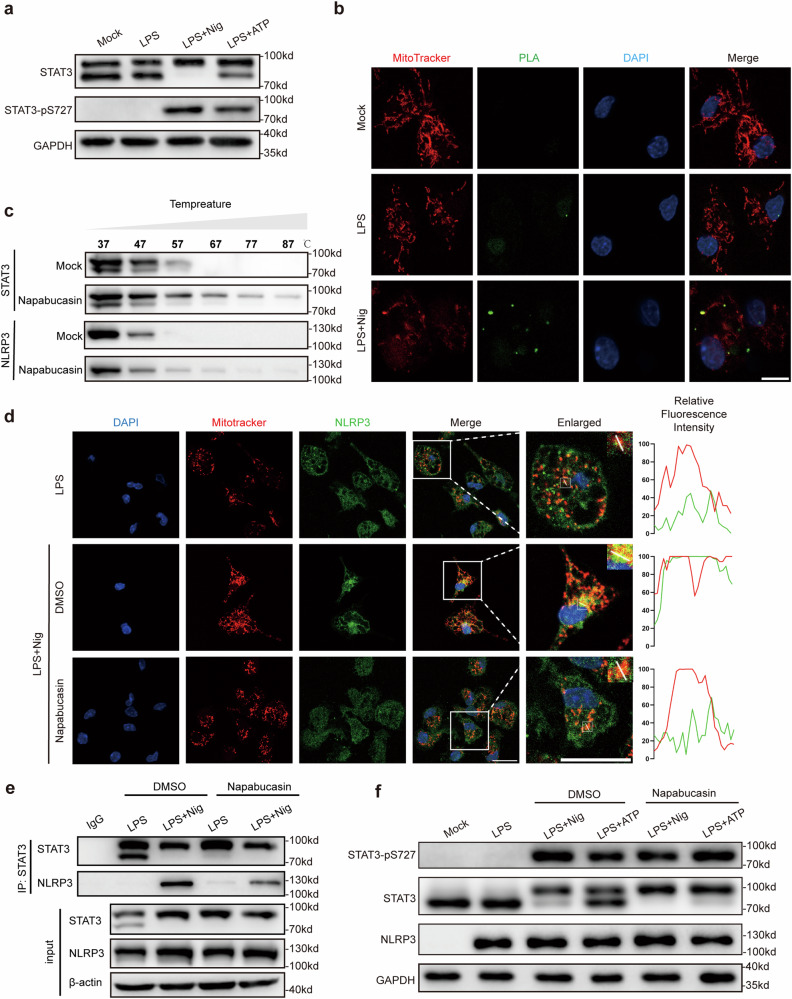
STAT3 transports NLRP3 to mitochondria. **a** Immunoblot analysis of cytoplasmic components using a cytoplasmic and nuclear fractionation kit after treatment with the corresponding stimuli described above. **b** The interaction between NLRP3 and STAT3 and the co-localization with mitochondria (red) in peritoneal macrophages were visualized by a PLA (green) and confocal microscopy. Scale bar: 10 μm. **c** Cellular thermal shift assay (CETSA) of STAT3 or NLRP3 with napabucasin (20 μM). **d** LPS-primed peritoneal macrophages were treated with the indicated stimuli. The colocalization of NLRP3 with mitochondria was visualized by immunofluorescence microscopy. Mitochondria were stained with MitoTracker CMXRos, NLRP3 was detected with Alexa Fluor 488, and cellular nuclei were stained with DAPI. The imaging data are representative of several images from three independent experiments. Scale bar: 20 μm. **e** Immunoprecipitation and immunoblot analysis of lysates from mouse peritoneal macrophages treated with the indicated stimuli with or without napabucasin (10 μM) prior to IP with an anti-STAT3 antibody and immunoblotting with an anti-NLRP3 antibody. **f** Cytoplasmic components were extracted with a cytoplasmic and nuclear extraction kit after stimulation as described above and were then analyzed by Western blotting. The results are presented as the means ± SDs, and representative photographs of three biologically independent experiments with similar results are shown.

Then, we further investigated the mechanism by which cytoplasmic STAT3 regulates NLRP3 inflammasome activation. Since STAT3 has been reported to regulate ETC function in mitochondria^19,26^, we speculated that the mitochondrial function of STAT3 contributes to NLRP3 inflammasome activation. We observed that treatment with the NLRP3 agonists nigericin and ATP increased Ser727 phosphorylation of STAT3 (Fig. 5a), which is a marker for STAT3 translocation to mitochondria^19,23^. Intriguingly, the STAT3-NLRP3 complex was colocalized with a mitochondrial marker (MitoTracker) (Fig. 5b). Thus, these results indicate that STAT3 can transport NLRP3 to mitochondria, as the translocation of NLRP3 to mitochondria has been reported to play a pivotal role in its activation. To verify this hypothesis, we sought to disrupt the interaction between STAT3 and NLRP3. Given that knockdown of STAT3 suppresses the expression of NLRP3, we cannot exclude the transcriptional effect of STAT3 on the regulation of NLRP3 inflammasome. Instead, we used napabucasin for the mechanistic study because it has been reported to bind to the SH2 domain of STAT3 and block STAT3 activity^21,27^. We first evaluated the potential of napabucasin to bind to STAT3 by a cellular thermal shift assay (CETSA)^28^, which detects the thermal stability of a protein upon ligand binding. Napabucasin increased the thermal stability of STAT3 but not that of NLRP3 (Fig. 5c), indicating that napabucasin indeed binds to STAT3. Thus, napabucasin was appropriate for use in subsequent mechanistic studies. The translocation of NLRP3 to mitochondria was significantly impaired in macrophages treated with napabucasin compared to control macrophages (Fig. 5d). Furthermore, napabucasin disrupted the interaction between STAT3 and NLRP3 during the assembly of the NLRP3 inflammasome after stimulation with LPS or Pam3CSK4, which activate the TLR4 and TLR1/2 pathways, respectively (Fig. 5e, Supplementary Fig. 2), but did not affect Ser727 phosphorylation of STAT3 (Fig. 5f). Taken together, these findings demonstrated that STAT3 mediates the translocation of NLRP3 to mitochondria for further activation of the NLRP3 inflammasome.

### Targeting STAT3 alleviates NLRP3-associated inflammation

**Fig. 6:**
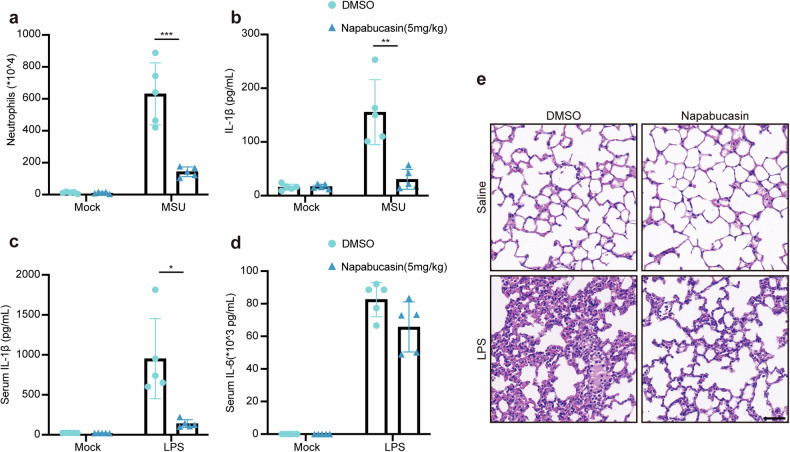
Targeting STAT3 alleviates NLRP3-associated inflammation. **a**, **b** Wild-type C57BL/6 mice were administered DMSO or napabucasin (5 mg/kg) via intraperitoneal (i.p.) injection 30 min before i.p. injection of MSU (2 mg per mouse) (*n* = 5 biologically independent mice) for 6 h. Quantification of neutrophils (**a**) and ELISA of IL-1β secretion (b) in the peritoneal lavage fluid. **c**–**e** Wild-type C57BL/6 mice were administered 20 mg/kg LPS via intraperitoneal (i.p.) injection (*n* = 5 biologically independent mice) for 8 h with or without napabucasin (5 mg/kg). ELISA results showing the serum concentrations of IL-1β (**c**) and IL-6 (**d**) and images of H&E-stained lung tissue sections (**e**). Scale bar: 50 μm. The data are representative of three independent experiments. The results are presented as the means ± SEMs. Statistical analyses were carried out via two-way ANOVA in (**a**–**d**). *P < 0.05, **P < 0.01 and ***P < 0.001.

Finally, we explored the potential of targeting STAT3 to alleviate NLRP3-associated inflammation. We established a model of peritonitis in mice by intraperitoneal (i.p.) injection of MSU, in which inflammation is NLRP3 inflammasome dependent. MSU challenge induced neutrophil infiltration and IL-1β secretion in peritoneal fluids, whereas napabucasin pretreatment reduced both of these parameters (Fig. 6a, b). In another LPS-induced endotoxin model, napabucasin exhibited anti-inflammatory effects by decreasing the serum IL-1β level and reducing lung tissue damage, alveolar edema and neutrophil infiltration compared with mice those in mice only challenged with LPS (Fig. 6c–e). Thus, targeting STAT3 could be a potential treatment strategy for NLRP3-associated inflammation.

## Discussion

**Fig. 7:**
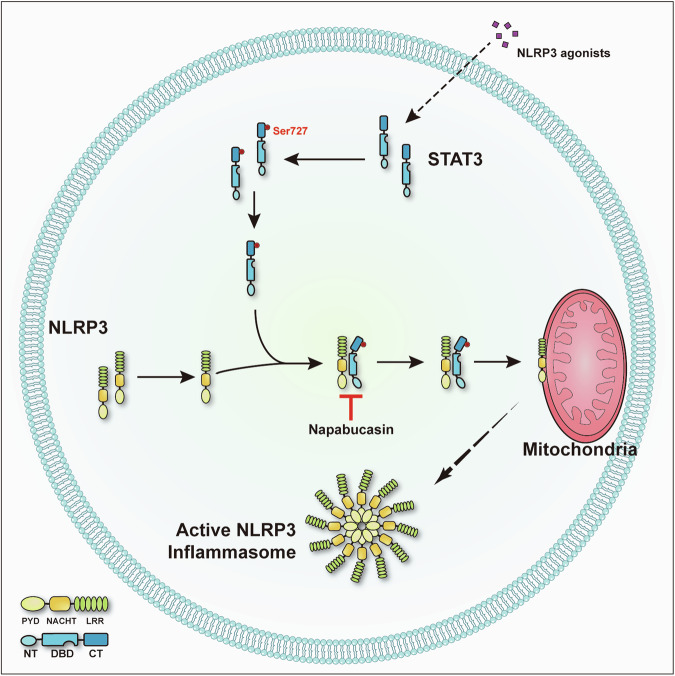
Model showing the role of STAT3 in NLRP3 inflammasome activation. STAT3 promotes NLRP3 inflammasome activation by mediating NLRP3 mitochondrial translocation, and this process is inhibited by napabucasin through disruption of the interaction between STAT3 and NLRP3.

Accumulating evidence has revealed that mitochondria play a crucial role in NLRP3 inflammasome activation via several mechanisms, including acting as scaffolds for the localization of NLRP3, releasing mitochondrial DNA (mtDNA) and mitochondrial ROS (mtROS) into the cytoplasm, and providing ATP for NLRP3 signaling, all of which contribute to the formation and activation of the NLRP3 inflammasome^29,30^. Although cardiolipin, MAVS and mitofusin-2 on the surface of mitochondria have been suggested to bind NLRP3 and recruit it to mitochondria^14–16^, how NLRP3 is translocated to mitochondria remains largely unknown. In this study, we demonstrated that STAT3 acts as a transporter for NLRP3 translocation to mitochondria (Fig. 7) and that blocking the interaction between STAT3 and NLRP3 by treatment with a STAT3 inhibitor substantially reduced NLRP3 inflammasome activation both in vitro and in vivo.

The knowledge of the translocation of NLRP3 to various organelles represents the greatest advancement in the field. Zhijian J. Chen’s group reported that diverse NLRP3 stimuli induce disassembly of the trans-Golgi network (TGN), after which the dispersed TGN (dTGN) serves as a scaffold for NLRP3 oligomerization and activation^31^. That work provided a new direction for exploring the underlying mechanism of NLRP3 activation. Subsequent studies revealed that BTK^32^, IKKβ^33^, and GSK3β^13^ are involved in regulating the location of NLRP3 in the dTGN. The most recent study showed, by using a live-cell multispectral time-lapse tracking system, that NLRP3 first translocates to mitochondria at approximately 10–15 min post stimulation and is subsequently recruited to the Golgi network^12^. This work, combined with previous studies, highlights the importance of NLRP3 translocation to mitochondria. In the present study, we also observed the translocation of NLRP3 to mitochondria upon stimulation of NLRP3, and disruption of NLRP3 mitochondrial translocation impaired the activation of the NLRP3 inflammasome. In mediating the translocation of NLRP3 to mitochondria, STAT3 mostly acts as a transporter by binding to NLRP3, which is different from the mechanism by which BTK, IKKβ and GSK3β in regulate the localization of NLRP3 to the dTGN. BTK^32^, IKKβ^33^ and GSK3β^13^ regulate the localization of NLRP3 to the dTGN in a manner dependent on their kinase function. For example, BTK phosphorylates specific tyrosine residues in the polybasic region of NLRP3, resulting in charge reversal in this region. This change promotes NLRP3 disassociation from the dTGN^32^. IKKβ and GSK3β have similar functions, but NLRP3 is not one of their substrates^13,33^. Previous studies have reported that Cardiolipin, MAVS and mitofusin-2 mediate the recruitment of NLRP3 to mitochondria by interacting with NLRP3^14–16^. However, these factors are located on the surface of mitochondria or the inner mitochondrial membrane; thus, how can they enable the translocation of cytosolic NLRP3 to mitochondria? Our results partially answer this question and provide a new explanation for the translocation of NLRP3 to mitochondria; i.e., that it is mediated by a transporter. Future work is needed to explore this dynamic process.

STAT3, a member of the STAT protein family, is a transcription factor that extensively participates in the regulation of acute and chronic inflammation, autoimmunity, metabolism, development and cancer progression^34–36^. It can be activated by various cytokines and growth factors, including IL-6, IL-10, IL-11, interferon, EGF, and HGF^37^. Upon activation, STAT3 is phosphorylated at two well-studied sites, Tyr705 and Ser727. When STAT3 undergoes phosphorylation at Tyr705, it is transported to the nucleus, where it can specifically bind to DNA for transcriptional activation^34^. The transcriptional regulation of NLRP3 and IL-1β by STAT3 has been investigated by several groups; however, no group has revealed a transcriptionally independent role for STAT3 in NLRP3 inflammasome regulation. By treatment with an inhibitor of STAT3 after LPS priming, we revealed the noncanonical function of STAT3 in NLRP3 inflammasome activation. This noncanonical function relies on Ser727 phosphorylation. When STAT3 undergoes phosphorylation at Ser727, it is transported to mitochondria and regulates ETC function^18,23^. Intriguingly, NLRP3 agonists can trigger Ser727 phosphorylation of STAT3; although the mechanism underlying this process is unknown, kinases responsible for Ser727 phosphorylation of STAT3 must exist. Several kinases, including EGF, PKC, JNK, ERK1, ERK2 and MAP kinases, have been reported to mediate Ser727 phosphorylation of STAT3^37–39^. Whether these kinases or unknown kinases are involved in this process still needs further investigation. In this study, we demonstrated that napabucasin binds to STAT3, but we cannot exclude the unknown effects of napabucasin on the NLRP3 inflammasome. In future studies, a series of STAT3 mutant mice which lost mitochondrial translocation effect needs to be investigated. In summary, we revealed a new role for STAT3 in regulating the location of NLRP3.

## Supplementary information

STAT3 promotes NLRP3 inflammasome activation by mediating NLRP3 mito- chondrial translocation

Supplementary Table 1

## References

[1] Cao, X. Self-regulation and cross-regulation of pattern-recognition receptor signalling in health and disease. *Nat. Rev. Immunol.* **16**, 35–50, 10.1038/nri.2015.8 (2016). 26711677 10.1038/nri.2015.8

[2] Brubaker, S. W., Bonham, K. S., Zanoni, I. & Kagan, J. C. Innate immune pattern recognition: a cell biological perspective. *Annu Rev. Immunol.* **33**, 257–290, 10.1146/annurev-immunol-032414-112240 (2015). 25581309 10.1146/annurev-immunol-032414-112240 PMC5146691

[3] Kawai, T. & Akira, S. The role of pattern-recognition receptors in innate immunity: update on Toll-like receptors. *Nat. Immunol.* **11**, 373–384, 10.1038/ni.1863 (2010). 20404851 10.1038/ni.1863

[4] Mantovani, A., Dinarello, C. A., Molgora, M. & Garlanda, C. Interleukin-1 and Related Cytokines in the Regulation of Inflammation and Immunity. *Immunity* **50**, 778–795, 10.1016/j.immuni.2019.03.012 (2019). 30995499 10.1016/j.immuni.2019.03.012 PMC7174020

[5] Broz, P., Pelegrin, P. & Shao, F. The gasdermins, a protein family executing cell death and inflammation. *Nat. Rev. Immunol.* **20**, 143–157, 10.1038/s41577-019-0228-2 (2020). 31690840 10.1038/s41577-019-0228-2

[6] Lamkanfi, M. & Dixit, V. M. Mechanisms and functions of inflammasomes. *Cell* **157**, 1013–1022, 10.1016/j.cell.2014.04.007 (2014). 24855941 10.1016/j.cell.2014.04.007

[7] Rathinam, V. A., Vanaja, S. K. & Fitzgerald, K. A. Regulation of inflammasome signaling. *Nat. Immunol.* **13**, 333–342, 10.1038/ni.2237 (2012). 22430786 10.1038/ni.2237 PMC3523703

[8] Swanson, K. V., Deng, M. & Ting, J. P. The NLRP3 inflammasome: molecular activation and regulation to therapeutics. *Nat. Rev. Immunol.* **19**, 477–489, 10.1038/s41577-019-0165-0 (2019). 31036962 10.1038/s41577-019-0165-0 PMC7807242

[9] Sharma, B. R. & Kanneganti, T. D. NLRP3 inflammasome in cancer and metabolic diseases. *Nat. Immunol.* **22**, 550–559, 10.1038/s41590-021-00886-5 (2021). 33707781 10.1038/s41590-021-00886-5 PMC8132572

[10] Mangan, M. S. J. et al. Targeting the NLRP3 inflammasome in inflammatory diseases. *Nat. Rev. Drug Discov.* **17**, 588–606, 10.1038/nrd.2018.97 (2018). 30026524 10.1038/nrd.2018.97

[11] Huang, Y., Xu, W. & Zhou, R. NLRP3 inflammasome activation and cell death. *Cell Mol. Immunol.* **18**, 2114–2127, 10.1038/s41423-021-00740-6 (2021). 34321623 10.1038/s41423-021-00740-6 PMC8429580

[12] Akbal, A. et al. How location and cellular signaling combine to activate the NLRP3 inflammasome. *Cell Mol. Immunol.* **19**, 1201–1214, 10.1038/s41423-022-00922-w (2022). 36127465 10.1038/s41423-022-00922-w PMC9622870

[13] Arumugam, S. et al. GSK3beta mediates the spatiotemporal dynamics of NLRP3 inflammasome activation. *Cell Death Differ.* **29**, 2060–2069, 10.1038/s41418-022-00997-y (2022). 35477991 10.1038/s41418-022-00997-y PMC9525599

[14] Iyer, S. S. et al. Mitochondrial cardiolipin is required for Nlrp3 inflammasome activation. *Immunity* **39**, 311–323, 10.1016/j.immuni.2013.08.001 (2013). 23954133 10.1016/j.immuni.2013.08.001 PMC3779285

[15] Subramanian, N., Natarajan, K., Clatworthy, M. R., Wang, Z. & Germain, R. N. The adaptor MAVS promotes NLRP3 mitochondrial localization and inflammasome activation. *Cell* **153**, 348–361, 10.1016/j.cell.2013.02.054 (2013). 23582325 10.1016/j.cell.2013.02.054 PMC3632354

[16] Tur, J. et al. Mitofusin 2 in Macrophages Links Mitochondrial ROS Production, Cytokine Release, Phagocytosis, Autophagy, and Bactericidal Activity. *Cell Rep.* **32**, 108079, 10.1016/j.celrep.2020.108079 (2020). 32846136 10.1016/j.celrep.2020.108079

[17] Yang, R. & Rincon, M. Mitochondrial Stat3, the Need for Design Thinking. *Int J. Biol. Sci.* **12**, 532–544, 10.7150/ijbs.15153 (2016). 27019635 10.7150/ijbs.15153 PMC4807418

[18] Hillmer, E. J., Zhang, H., Li, H. S. & Watowich, S. S. STAT3 signaling in immunity. *Cytokine Growth Factor Rev.* **31**, 1–15, 10.1016/j.cytogfr.2016.05.001 (2016). 27185365 10.1016/j.cytogfr.2016.05.001 PMC5050093

[19] Wegrzyn, J. et al. Function of mitochondrial Stat3 in cellular respiration. *Science* **323**, 793–797, 10.1126/science.1164551 (2009). 19131594 10.1126/science.1164551 PMC2758306

[20] Wang, D. et al. YAP promotes the activation of NLRP3 inflammasome via blocking K27-linked polyubiquitination of NLRP3. *Nat. Commun.* **12**, 2674, 10.1038/s41467-021-22987-3 (2021). 33976226 10.1038/s41467-021-22987-3 PMC8113592

[21] Li, Y. et al. Suppression of cancer relapse and metastasis by inhibiting cancer stemness. *Proc. Natl Acad. Sci. USA* **112**, 1839–1844, 10.1073/pnas.1424171112 (2015). 25605917 10.1073/pnas.1424171112 PMC4330785

[22] Jonker, D. J. et al. Napabucasin versus placebo in refractory advanced colorectal cancer: a randomised phase 3 trial. *Lancet Gastroenterol. Hepatol.* **3**, 263–270, 10.1016/S2468-1253(18)30009-8 (2018). 29397354 10.1016/S2468-1253(18)30009-8

[23] Balic, J. J. et al. STAT3 serine phosphorylation is required for TLR4 metabolic reprogramming and IL-1beta expression. *Nat. Commun.* **11**, 3816, 10.1038/s41467-020-17669-5 (2020). 32732870 10.1038/s41467-020-17669-5 PMC7393113

[24] Samavati, L. et al. STAT3 tyrosine phosphorylation is critical for interleukin 1 beta and interleukin-6 production in response to lipopolysaccharide and live bacteria. *Mol. Immunol.* **46**, 1867–1877, 10.1016/j.molimm.2009.02.018 (2009). 19299019 10.1016/j.molimm.2009.02.018

[25] Zhu, L. et al. STAT3/Mitophagy Axis Coordinates Macrophage NLRP3 Inflammasome Activation and Inflammatory Bone Loss. *J. Bone Min. Res* **38**, 335–353, 10.1002/jbmr.4756 (2023). 10.1002/jbmr.4756 36502520

[26] Carbognin, E., Betto, R. M., Soriano, M. E., Smith, A. G. & Martello, G. Stat3 promotes mitochondrial transcription and oxidative respiration during maintenance and induction of naive pluripotency. *EMBO J.* **35**, 618–634, 10.15252/embj.201592629 (2016). 26903601 10.15252/embj.201592629 PMC4801951

[27] Tahara, T. et al. STAT3 inhibitory activity of naphthoquinones isolated from Tabebuia avellanedae. *Bioorg. Med Chem.* **28**, 115347, 10.1016/j.bmc.2020.115347 (2020). 32044231 10.1016/j.bmc.2020.115347

[28] Peng, Y. et al. Lovastatin inhibits Toll-like receptor 4 signaling in microglia by targeting its co-receptor myeloid differentiation protein 2 and attenuates neuropathic pain. *Brain Behav. Immun.* **82**, 432–444, 10.1016/j.bbi.2019.09.013 (2019). 31542403 10.1016/j.bbi.2019.09.013

[29] Xian, H. et al. Oxidized DNA fragments exit mitochondria via mPTP- and VDAC-dependent channels to activate NLRP3 inflammasome and interferon signaling. *Immunity* **55**, 1370–1385.e1378, 10.1016/j.immuni.2022.06.007 (2022). 35835107 10.1016/j.immuni.2022.06.007 PMC9378606

[30] Xian, H. et al. Metformin inhibition of mitochondrial ATP and DNA synthesis abrogates NLRP3 inflammasome activation and pulmonary inflammation. *Immunity* **54**, 1463–1477.e1411, 10.1016/j.immuni.2021.05.004 (2021). 34115964 10.1016/j.immuni.2021.05.004 PMC8189765

[31] Chen, J. & Chen, Z. J. PtdIns4P on dispersed trans-Golgi network mediates NLRP3 inflammasome activation. *Nature* **564**, 71–76, 10.1038/s41586-018-0761-3 (2018). 30487600 10.1038/s41586-018-0761-3 PMC9402428

[32] Bittner, Z. A. et al. BTK operates a phospho-tyrosine switch to regulate NLRP3 inflammasome activity. *J. Exp. Med.* **218**, 10.1084/jem.20201656 (2021). 10.1084/jem.20201656 PMC8480672 34554188

[33] Nanda, S. K., Prescott, A. R., Figueras-Vadillo, C. & Cohen, P. IKKbeta is required for the formation of the NLRP3 inflammasome. *EMBO Rep.* **22**, e50743, 10.15252/embr.202050743 (2021). 34403206 10.15252/embr.202050743 PMC8490994

[34] Reich, N. C. & Liu, L. Tracking STAT nuclear traffic. *Nat. Rev. Immunol.* **6**, 602–612, 10.1038/nri1885 (2006). 16868551 10.1038/nri1885

[35] Zou, S. et al. Targeting STAT3 in Cancer Immunotherapy. *Mol. Cancer* **19**, 145, 10.1186/s12943-020-01258-7 (2020). 32972405 10.1186/s12943-020-01258-7 PMC7513516

[36] Johnson, D. E., O’Keefe, R. A. & Grandis, J. R. Targeting the IL-6/JAK/STAT3 signalling axis in cancer. *Nat. Rev. Clin. Oncol.* **15**, 234–248, 10.1038/nrclinonc.2018.8 (2018). 29405201 10.1038/nrclinonc.2018.8 PMC5858971

[37] Yu, H., Pardoll, D. & Jove, R. STATs in cancer inflammation and immunity: a leading role for STAT3. *Nat. Rev. Cancer* **9**, 798–809, 10.1038/nrc2734 (2009). 19851315 10.1038/nrc2734 PMC4856025

[38] Sgrignani, J. et al. Structural Biology of STAT3 and Its Implications for Anticancer Therapies Development. *Int. J. Mol. Sci.* **19**, 10.3390/ijms19061591 (2018). 10.3390/ijms19061591 PMC6032208 29843450

[39] Macias, E., Rao, D., Carbajal, S., Kiguchi, K. & DiGiovanni, J. Stat3 binds to mtDNA and regulates mitochondrial gene expression in keratinocytes. *J. Invest Dermatol.* **134**, 1971–1980, 10.1038/jid.2014.68 (2014). 24496235 10.1038/jid.2014.68 PMC4057971

