# Author Correction: STAT3 promotes NLRP3 inflammasome activation by mediating NLRP3 mitochondrial translocation

**DOI:** 10.1038/s12276-024-01327-7

**Published:** 2024-09-27

**Authors:** Ling Luo, Fupeng Wang, Xueming Xu, Mingliang Ma, Guangyan Kuang, Yening Zhang, Dan Wang, Wei Li, Ningjie Zhang, Kai Zhao

**Affiliations:** 1 Department of Hematology and Critical Care Medicine, Third Xiangya Hospital, Central South University, Changsha, Hunan Province 410000 P PR China; 2 Department of Dermatology, Third Xiangya Hospital, Central South University, Changsha, Hunan Province 410000 P PR China; 3 Department of Rheumatology, First Affiliated Hospital of Zhengzhou University, Zhengzhou, Henan Province 450000 P PR China; 4 Department of Blood Transfusion, Second Xiangya Hospital, Central South University, Changsha, Hunan Province 410000 P PR China; 5 Key Laboratory of Sepsis Translational Medicine of Hunan, Central South University, Changsha, Hunan Province 410000 P PR China

**Keywords:** Cell death and immune response, Cell signalling

Correction to: *Experimental & Molecular Medicine* 10.1038/s12276-024-01298-9, published online 2 September 2024

After online publication of this article, the authors noticed an error in the “Mice” section in “Materials and methods”.

“The animal experiments were conducted in accordance with the Institutional Animal Care and Use Committee of Central South University.”

The correct statement of this article should have read as below.

“The animal experiments were conducted in accordance with the Institutional Animal Care and Use Committee of Central South University (CSU-2022-0657).”

The authors apologize for any inconvenience caused.

The original article has been corrected.

