# Supplementary material for: STAT3 promotes NLRP3 inflammasome activation by mediating NLRP3 mitochondrial translocation
Source: Exp Mol Med. 2024 Sep 2;56(9):1980–90. doi: 10.1038/s12276-024-01298-9 (PMC11446920; doi:10.1038/s12276-024-01298-9)
Supplement: Supplementary file 1 — STAT3 promotes NLRP3 inflammasome activation by mediating NLRP3 mito- chondrial translocation [file 12276_2024_1298_MOESM1_ESM.pdf]

Supplementary Figure 1

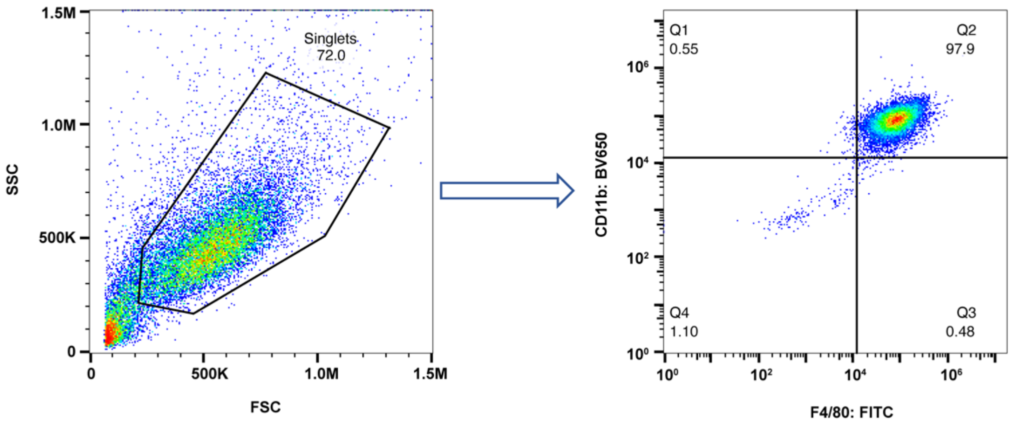

Supplementary Fig. 1: Analysis of the purity of macrophages in peritoneal cells. Three days after the injection of thioglycollate, mouse primary macrophages were extracted by peritoneal lavage with 1640 medium. Macrophages were labeled with F4/80 and CD11b, and the proportion of extracted macrophages was detected by flow cytometry.

Supplementary Figure 2

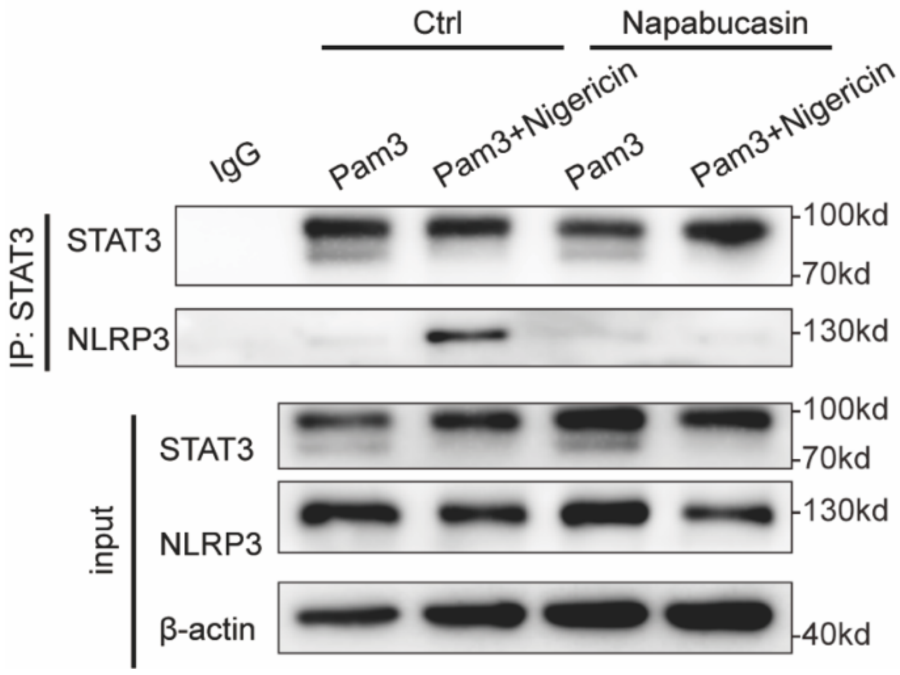

Supplementary Fig. 2: Analysis of the blocking effect of Napabucasin on STAT3 and NLRP3.

Peritoneal macrophages were treated with Pam3 (Pam3CSK4, 1 μg/mL, 3h) then followed DMSO or Napabucasin (10 μM, 30min) and cells were continued to be stimulated with Nigericin (10 μM, 1h). the interaction between STAT3 and NLRP3 was determined by Immunoprecipitation.
